# Scale‐down studies for the scale‐up of a recombinant *Corynebacterium glutamicum* fed‐batch fermentation: loss of homogeneity leads to lower levels of cadaverine production

**DOI:** 10.1002/jctb.6248

**Published:** 2019-11-21

**Authors:** Williams Olughu, Alvin Nienow, Chris Hewitt, Chris Rielly

**Affiliations:** ^1^ Department of Chemical Engineering Loughborough University Loughborough UK; ^2^ Ipsen Biopharma Ltd Wrexham UK; ^3^ School of Chemical Engineering University of Birmingham Birmingham UK; ^4^ School of Life and Health Sciences Aston University Birmingham UK

**Keywords:** biochemical engineering, bioprocesses, biotechnology, fermentation, industrial biotechnology, process development

## Abstract

**BACKGROUND:**

The loss of efficiency and performance of bioprocesses on scale‐up is well known, but not fully understood. This work addresses this problem, by studying the effect of some fermentation gradients (pH, glucose and oxygen) that occur at the larger scale in a bench‐scale two‐compartment reactor [plug flow reactor (PFR) + stirred tank reactor (STR)] using the cadaverine‐producing recombinant *Corynebacterium glutamicum* DM1945 Δact3 Ptuf‐ldcC_OPT. The new scale‐down strategy developed here studied the effect of increasing the magnitude of fermentation gradients by considering not only the average cell residence time in the PFR (*τ*_PFR_), but also the mean frequency at which the bacterial cells entered the PFR (*f*_m_) section of the two‐compartment reactor.

**RESULTS:**

On implementing this strategy the cadaverine production decreased on average by 26%, 49% and 59% when the *τ*_PFR_ was increased from 1 to 2 min and then 5 min respectively compared to the control fermentation. The carbon dioxide productivity was highest (3.1‐fold that of the control) at a *τ*_PFR_ of 5 min, but no losses were observed in biomass production. However, the population of viable but non‐culturable cells increased as the magnitude of fermentation gradients was increased. The new scale‐down approach was also shown to have a bigger impact on fermentation performance than the traditional one.

**CONCLUSION:**

This study demonstrated that *C. glutamicum* DM1945 Δact3 Ptuf‐ldcC_OPT physiological response was a function of the magnitude of fermentation gradients simulated. The adaptations of a bacterial cell within a heterogeneous environment ultimately result in losses in fermentation productivity as observed here. © 2019 The Authors. *Journal of Chemical Technology & Biotechnology* published by John Wiley & Sons Ltd on behalf of Society of Chemical Industry.

**NOMENCLATURE**
*f*_m_: mean frequency at which the bacterial cells entered the plug flow reactor
*F*_0_: feeding flow rate
*Q*_r_: volumetric recirculation flow rate
*S*: feed glucose concentration
*t*: time
*V*_0_: working volume of the stirred tank reactor
*X*_0_: dry cell weight
*Y*_*x*/*s*_: yield of biomass on glucose
*μ*: specific growth rate
*τ*_PFR_: cell mean residence time in the plug flow reactor
*τ*_STR_: cell mean residence time in the stirred tank reactor

## INTRODUCTION

One of the biggest challenges facing civilization of the twenty‐first century is the unprecedented climate change resulting from global warming, a change mainly driven by anthropogenic activities.^1^ The biorefinery complex is seen as a more benign means of producing useful products/medicines for humans. More recently, its implementation has been driven by the need to reduce the overreliance on fossilized fuel and increase sustainability.^2^, ^3^ However, before biorefineries become a reality, a proper understanding of the large‐scale fermentation environment is crucial to develop bioprocesses with productivities at par with or that even surpassing those of the petrol‐chemical industry.

The need for a viable biorefinery complex has driven the search for biologically produced compounds that can be easily used as precursors to make important commercial product(s). One such compound is cadaverine (1,5‐diaminopentane), a platform chemical of the future which can be used in the production of various biopolymer materials, such as polyamide‐5.4, which has a high fatigue resistance, high melting point and low density.^4^ The microorganism of choice was *Corynebacterium glutamicum* because of its natural link to cadaverine; as a commercial producer of lysine, it is only a decarboxylation step away from yielding cadaverine, see Eqn (1) 
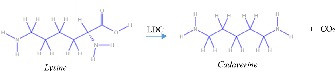


The lysine decarboxylase (LDC) enzyme converts lysine to cadaverine. Hence, *C. glutamicum* with the gene insert that activates the LDC enzyme complex results in the production of cadaverine. Also, *C. glutamicum's* excellent record of safety from its extensive use in commercial amino acid production was an essential factor in its selection.^5^

This work focuses on quantifying the effect of the large‐scale heterogeneous environment on a recombinant *C. glutamicum* cadaverine producing strain; its physiological response was studied using both single‐cell and bulk‐cell assays.

Understanding the heterogeneous large‐scale fed‐batch fermentation process remains an ongoing problem for the bioprocess industry. Currently, the scale‐up environment is typically an afterthought in bioprocess development. This mindset results in the persistent issues of decline in productivity and product quality that plagues most large‐scale fermentations.^6^, ^7^, ^8^, ^9^

The large‐scale fermentation environment is fundamentally different (due to the increased likelihood of chemical and physical gradients formation) to the small‐scale fermenter, where bioprocess development occurs.^10^, ^11^ For example, in the commercially ubiquitous large‐scale fed‐batch fermentation, there exist localized high feed concentration zones, large hydrostatic pressure differences resulting in changes in gas solubilities and inefficient mixing, which are some of the factors that create a non‐ideal environment for cells to grow and produce product.^12^, ^13^

Useful tools have been developed over the years to perform low cost, small‐scale tests to gain a better understanding of the effects of spatial gradients in large‐scale fermenter operations. The scale‐down reactor (SDR) is one such tool, which stems from compartmentalizing regions of gradients (for example, the high substrate concentration zone near the feed addition area) that are known to exist at the large‐scale.^9^, ^10^ The well characterized 30 m^3^ stirred tank reactor (STR) of Enfors *et al*.^14^ was mimicked using a two‐compartment SDR [STR + PFR (plug flow reactor)] to study the effect of three typical chemical/fermentation gradients [dissolved oxygen (DO), glucose and pH] which exist at the large scale. However, the SDR strategy implemented in this study was different, as is explained later.

**Figure 1:**
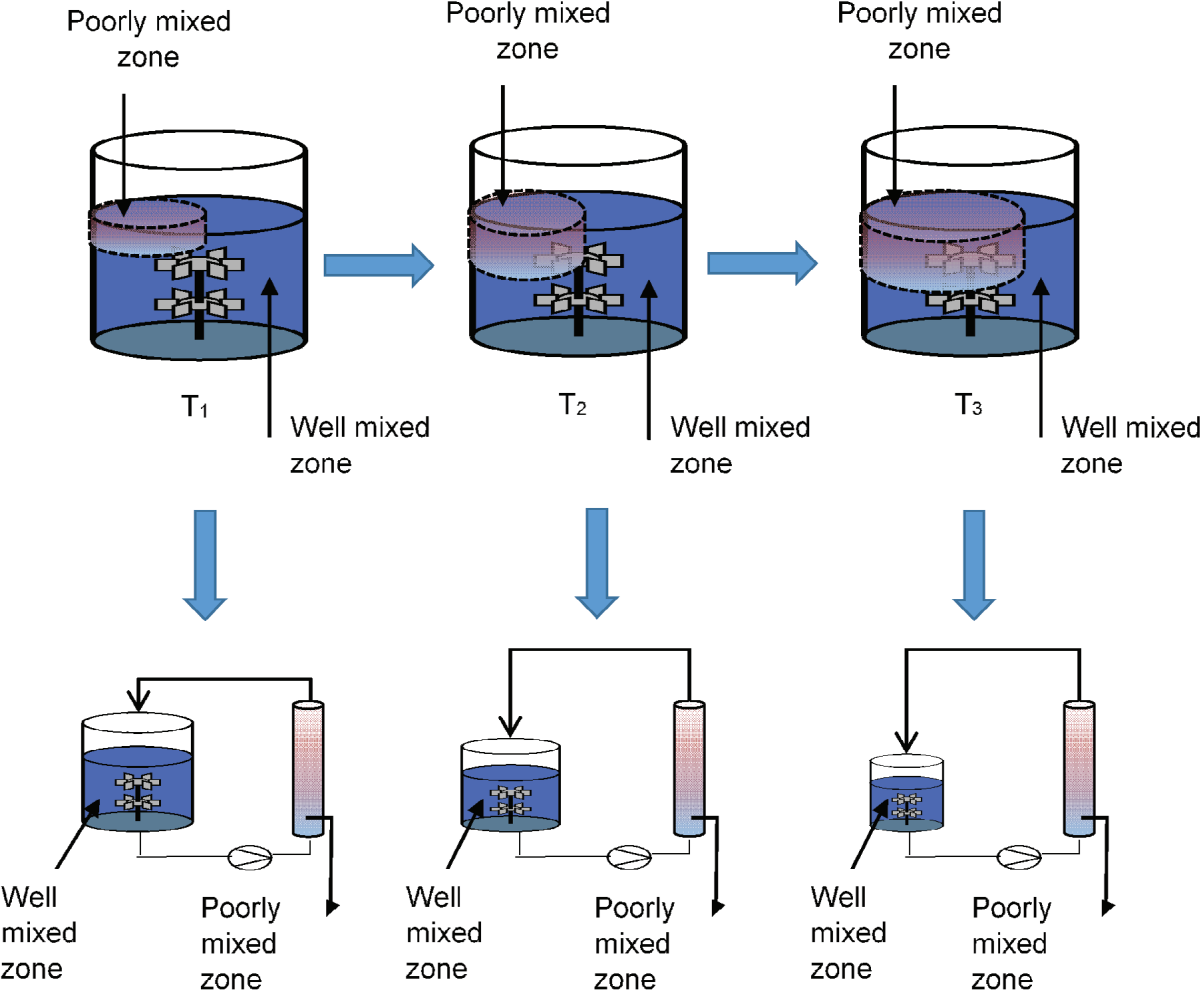
The two‐compartment scale‐down models adapted to mimic changes in the dead zone proportions of a high cell density large‐scale process due to declining mixing efficiency. T_1_, T_2_ and T_3_ represent increasing timeframes during a fed‐batch fermentation process

Typically, when investigators study the effect of large‐scale fed‐batch fermentation gradients in the laboratory using SDRs, little or no consideration is given to its dynamic environment. The impact of increasing broth viscosity, which sometimes occurs in the latter part of a fed‐batch process is paid little attention. This increase in viscosity results from a high cell biomass concentration, changes in cell morphology and polymer product formation.^13^ Thus, if during the latter stage of a high cell density fed‐batch fermentation, the dynamic broth viscosity exceeds 50 mPa s, a transitional flow regime dominates.^15^ Such a situation reduces the heat and mass transfer efficiency of the fermenting vessel and thereby increases the spatial and temporal occurrence of dead zones. These effects increase the magnitude of fermentation gradients over the course of a fed‐batch process (*T*_1_ to *T*_3_), as illustrated in Fig. 1.

Figure 1 indicates that the proportion of the poorly mixed zone to the well‐mixed region increases as the mixing time increases (note that time *T*_1_ < *T*_2_ < *T*_3_). The strategy to mimic the effect of increasing the mixing time using the two‐compartment SDR is also shown in Fig. 1. The progression from *T*_1_ to *T*_3_ was simulated by reducing the volume in the STR while the PFR volume stayed constant. This changing ratio of the poorly mixed zone to the well‐mixed region is thought to be a closer reflection to increasing the magnitude of fermentation gradients when the mean residence time in the PFR section was increased. Thus, the current work uses a series of SDR simulations to understand the effects of changing the relative volumes of the STR and PFR, as well as studying the impact of the mean residence time in the PFR section, on the product formation rate.

## MATERIALS AND METHODS

All fermentations here started as a batch process up until the dry cell weight (DCW) reached 1 g L^−1^, at which point the fed‐batch stage was started. The chemicals used were all of reagent grade.

*Corynebacterium glutamicum* DM1945 Δact3 Ptuf‐ldcC_OPT (abbreviated, *C. glutamicum* DM1945x3) was genetically modified by insertion of the lysine decarboxylase ldcC gene from *Escherichia coli*, a gene responsible for the decarboxylation of intracellular lysine to extracellular cadaverine.^16^

The inoculation medium was 30 g L^−1^ tryptone soya broth (TSB, Oxoid, Basingstoke, UK) in deionized water. The growth medium composition used in the small‐scale bioreactor for the batch stage comprised glucose, 10 g L^−1^; (NH_4_)_2_SO_4_, 20 g L^−1^; urea, 5 g L^−1^; MgSO_4_·7H_2_O, 0.13 g L^−1^; KH_2_PO_4_, 1 g L^−1^; K_2_HPO_4_, 1 g L^−1^; *protocatechuic acid, 1 mL L^−1^; *CaCl_2_·2H_2_O, 1 mL L^−1^; *Biotin, 1 mL L^−1^; *trace elements, 1 mL L^−1^ (*indicates filter sterile stock solutions made before addition to autoclavable medium components). The 0.22 μm filtered stock solutions consisted of protocatechuic acid, 30 g L^−1^ in 1 mol L^−1^ of NaOH; CaCl_2_·2H_2_O, 13.25 g L^−1^; Biotin, 0.2 g L^−1^ in 1 mol L^−1^ of NaOH. The 0.22 μm filtered trace elements composition was FeSO_4_·7H_2_O, 10 g L^−1^; MnSO_4_·H_2_O, 10 g L^−1^; ZnSO_4_·7H_2_O, 1 g L^−1^; CuSO_4_·5H_2_O, 0.313 g L^−1^; NiCl_2_·6H_2_O, 0.02 g L^−1^, each of which was dissolved in 1 mol L^−1^ HCl.

For the fed‐batch addition, the medium components was composed of glucose, 620 g L^−1^; (NH_4_)_2_SO_4_, 40 g L^−1^; urea, 10 g L^−1^; MgSO_4_·7H_2_O, 0.25 g L^−1^; KH_2_PO_4_, 2 g L^−1^; K_2_HPO_4_, 2 g L^−1^; protocatechuic acid, 2 mL L^−1^; CaCl_2_·2H_2_O, 2 mL L^−1^; Biotin, 2 mL L^−1^; trace elements, 2 mL L^−1^. Unless otherwise stated the earlier mentioned reagents were dissolved in deionized water, also the glucose was heat sterilized separately to avoid the Maillard reaction.

*Corynebacterium glutamicum* DM1945x3 working cell bank was stored at −80 °C in the Microbanks™ preservation phials (Pro‐Lab Diagnostics, Birkenhead, UK). The initial inoculum was reconstituted by streaking a bead from the cell bank onto a tryptone soya agar (TSA) filled plate (Oxoid), and incubated for 48 h at 30 °C. A viable colony was then selected and transferred to an unbaffled Erlenmeyer flask containing 150 mL of sterile TSB medium. This inoculated flask was then cultivated overnight (13 h) in an Innova® incubator shaker (Eppendorf, Hauppauge, NY, USA) at 170 rpm, and 30 °C before expansion into the 5 L STR.

**Figure 2:**
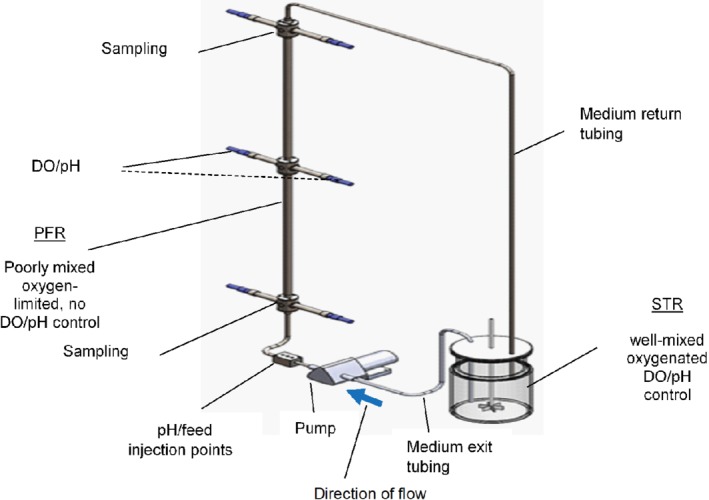
Schematic representing the scale‐down reactor (SDR) experimental rig

This study used a two‐compartment SDR setup comprising a PFR connected in series with an STR. The STR was a standard jacketed 5 L Sartorius Stedim, Biostat B plus reactor, fitted with two Rushton impellers (power number, 4.5). The vessel had a height, 28.2 cm, diameter, 15 cm, the distance from vessel base to lower impeller, 4.7 cm and the length between impellers, 7.6 cm. The SDR strategy here varied the STR volume from 3, 1.5 and 0.6 L as the cell mean residence time in the PFR (*τ*_PFR_) was increased from 1, 2 and 5 min, respectively, by reducing the recirculation pump (illustrated in Fig. 2) flowrate. The *τ*_PFR_ was increased to mimic a worsening mixing situation at the large scale, and as thus exposing the *C. glutamicum* DM1945x3 cells to increasing fermentation gradients (pH, glucose, and DO), while their physiological response was observed. The stainless‐steel PFR had a length of 165 cm and an internal diameter of 1.5 cm. There were 96 radial mixing elements inserted along its length to enhance plug flow characteristics. The PFR total working volume was 320 mL, which was fixed in all cases; Fig. 2 shows a schematic of the SDR setup.

When the working volume in the STR was varied, it was guided by the relationship shown in Eqn (2). Although this strategy decreased the STR volume as the *τ*_PFR_ was increased, the mean frequency at which the cells entered the PFR (*f*_m_) was kept constant at 0.0018 s^−1^. This meant that increasing the *τ*_PFR_ did not increase the cell mean residence time in the STR (*τ*_STR_), hence cells might not have the additional time in the ideal STR environment to recover.

(2) $$f_{m}=\frac{Q_{r}}{V_{0}}$$

where, *Q*_r_ represents the volumetric recirculation flow rate (mL s^−1^) and *V*_0_ the working volume of the STR (in millilitres).

The batch phase of the fermentation lasted for 8 h after inoculation. After which, feeding and recirculation started and at this point, the biomass concentration was *ca* 1 g L^−1^ DCW. The total fermentation time in all simulations studied was 39 h. All fermentations were carried out at a temperature of 30 °C; the STR was sparged with air constantly at a rate of 1.5 vvm and a fixed agitation rate of 800 rpm. Towards the latter stages of fermentation at high cell densities (DCW > 25 g L^−1^), the dissolved oxygen tension (DOT) was maintained ≥ 40% of saturation by gas blending to vary the oxygen mass fraction, while the pH was controlled at 7 by the addition of 4 mol L^−1^ NH_4_OH on demand.

In contrast, in the PFR, the DOT, pH and temperature probes only monitored operating conditions. There was no process control in the PFR section of the SDR (see Fig. 2). However, in the SDR runs where pH gradients were simulated, the rate of base addition in the PFR was a function of the pH measured and controlled at the set point in the STR, as a result of the cells metabolic activity throughout the SDR.

Equation (3) was used to calculate the exponential feeding flow rate into the STR from the end of the batch phase.

(3) $$F_{0}=\frac{\mu }{S*Y_{x/s}}*\left(X_{0}V_{0}\right)*e^{\mu t}$$

where *F*_0_ represents the feeding flow rate at time, *t* (in L h^−1^), *μ* is the intended specific growth rate (in h^−1^), *S* the feed glucose concentration (in g L^−1^), *X*_0_ = DCW at *t* (in g L^−1^), *V*_0_ is the working volume of STR at *t* (in litres), *t* the time (in hours), *Y*_*x*/*s*_ is the yield of biomass on glucose (in g g^−1^).

The exponential feeding flow rate profile of Eqn (3) was maintained until the termination of the fermentation, with a *μ* of approximately 0.1 h^−1^ held throughout. The low *μ* was adopted to minimize the formation of side products, such as lactate and dihydroxyacetone, via the overflow metabolic pathway.^17^

The BD FACSJazz™ cell sorter (BD Biosciences, Franklin Lakes, NJ, USA) flow cytometer was used to evaluate the cytoplasmic membrane potential and integrity of *C. glutamicum* DM1945x3 (an indication of cell viability). It had three laser light sources, blue (488 nm at 80 mW), red (640 nm at 50 mW) and violet (405 nm at 50 mW). However, only the blue laser was used for this work, as it was sufficient to excite the two fluorochromes adopted here. The fluorochromes were propidium iodide (PI) and DiBAC4^3^ (bis‐(1,3‐dibutylbarbituric acid)trimethine oxonol) (Oxonol). These fluorochromes are usually excluded from healthy cells with intact and fully polarized cytoplasmic membranes.^18^ PI binds to the DNA of a cell with a breached membrane barrier, while Oxonol is a lipophilic, anionic dye, which accumulates intracellularly provided the cell is ineffective at transporting it out of the cytoplasmic membrane, and thus it is deemed to be depolarized. The stock solutions of 3 μmol L^−1^ Oxonol in dimethyl sulphoxide and 20 mmol L^−1^ of PI in deionized water were used for this analysis. From working solutions, 0.2 μL Oxonol and 0.1 μL PI were added to a diluted 1 mL solution (*ca* 10^6^ cells mL^‐1^) of cell suspension in filtered phosphate‐buffered saline (PBS). This solution was then mixed and incubated at room temperature in the dark for 10 min, before injection to the flow cytometer.

The colony‐forming unit (CFU) was measured by pouring a sterile TSA (Oxoid) solution into Petri dishes, which held 100 μL aliquots of diluted cell suspensions ranging from 10^−5^ to 10^−7^. This mixture was then gently stirred and incubated at 30 °C for 48 h before counting the visible colonies under a magnifying glass.

The growth of cells was monitored by quantifying the concentration of the DCW per litre of fermentation broth. These values were derived by centrifuging a 5 mL sample at 4000 × *g* and 4 °C for 10 min; the resulting precipitate was then washed and re‐centrifuged under the same conditions. After that, it was allowed to dry at 80 °C for 48 h before weighing.

A high‐performance liquid chromatography (HPLC) series 200 (Perkin Elmer, Waltham, MA, USA) fitted with a 265, 4.8 nm UV‐visible detector was used to determine cadaverine concentration. A pre‐column Raptor™ARC‐18 (Restek Corporation, Bellefonte, PA, USA) derivatization reaction was needed for cadaverine quantification. Furthermore, 150 μL of 30 mmol L^−1^ 9‐fluorenylmethoxycarbonyl chloride (FMOC) in 150 μL acetonitrile and 150 μL of 0.2 mol L^−1^ borate buffer were reacted with a 150 μL filtered (0.22 μm filter) fermentation sample. The two mobile phases were used, the first was a mixture of 0.1% formic acid and 20 mmol L^−1^ ammonium formate in water (A) and the second was 0.1% formic acid and 10 mmol L^−1^ ammonium formate in 90:10 acetonitrile/water (B).

The Tandem gas analyser (Magellan BioTech, Borehamwood, UK) was used to measure the oxygen and carbon dioxide (CO_2_) composition of the air in and out of the fermenting vessel. The inlet and exit air were filtered via a 0.22 μm filter before being diverted to the analyser.

## RESULTS AND DISCUSSION

The few investigators who have studied the effect of increasing large‐scale fermentation gradients in *C. glutamicum* have simulated it only by increasing the cell mean residence time in the poorly mixed section (PFR or STR) of the scale‐down model.^19^, ^20^, ^21^, ^22^ This scale‐down model was an attempt to mimic the relatively poor homogeneity on the large‐scale compared to the small‐scale, because of the increasing mixing time with many organisms (see for example the studies in the references,^7^, ^10^, ^12^, ^13^, ^16^, ^17^). Thus, in these past STR + PFR models, a worsening mixing situation is typically mimicked by only increasing *τ*_*P*FR_. Such a strategy however does not consider that by only increasing the *τ*_PFR_, (poorly‐mixed section), there is also a proportional increase in the *τ*_*STR*_ (well‐mixed section). This results in more cells spending more time in the well‐mixed section of the SDR, which may increase the chance of recovery before being recirculated back to the poorly‐mixed section. Hence, there may be attenuation of any net loss in productivity from the simulated fermentation gradient. Also, there is an increased buffer effect, due to the increased proportion of the cells in the well‐mixed section to the poorly‐mixed section, when only *τ*_PFR_ is increased. These factors may explain the insignificant physiological change observed in some past scale‐down studies of *C. glutamicum*,^20^, ^22^ though significant impacts have been noted with other microorganisms, notably yeast,^10^ *Bacillus subtillis* 7 and *E. coli*.^6^, ^16^, ^17^

Alternatively, to accurately compare the effect of deteriorating mixing conditions within the same biological system in SDRs, the volumetric proportions of its different compartments must be varied in tandem with the cell mean residence time. Here, the strategy of keeping the mean frequency (*f*_m_) at which the cells entered the PFR constant regardless of the selected *τ*_PFR_ was adopted. Consequently, the volume of the STR was adjusted accordingly to achieve this, as shown in Table 1.

**Table 1:** Experimental overview of the scale‐down reactors (SDRs) investigated, stirred tank reactor (STR) only represents the control

Simulation	Glucose inlet	pH inlet	Air inlet	STR volume (L)	PFR volume (L)	*τ*_PFR_ (min)	*τ*_STR_ (min)	*f*_m_ (s)	Fermentation gradient studied
STR only	STR	STR	STR	3	N/A	N/A	N/A	N/A	N/A
SDR 1	STR	STR	STR	3	0.3	1	10	0.0018	Oxygen limitation
SDR 2	STR	STR	STR	1.5	0.3	2	10	0.0018	
SDR 3	STR	STR	STR	0.6	0.3	5	10	0.0018	
SDR 4	PFR	STR	STR	3	0.3	1	10	0.0018	High glucose concentration and oxygen limitation
SDR 5	PFR	STR	STR	1.5	0.3	2	10	0.0018	
SDR 6	PFR	STR	STR	0.6	0.3	5	10	0.0018	
SDR 7	STR	PFR	STR	3	0.3	1	10	0.0018	High pH oscillations and oxygen limitations
SDR 8	STR	PFR	STR	1.5	0.3	2	10	0.0018	
SDR 9	STR	PFR	STR	0.6	0.3	5	10	0.0018	
SDR 10	PFR	PFR	STR	3	0.3	1	10	0.0018	High pH oscillations, High glucose concentration and oxygen limitations
SDR 11	PFR	PFR	STR	1.5	0.3	2	10	0.0018	
SDR 12	PFR	PFR	STR	0.6	0.3	5	10	0.0018	
SDR 13	PFR	PFR	STR	3	0.3	2	20	0.0009	
SDR 14	PFR	PFR	STR	3	0.3	5	50	0.0004	

N/A, not applicable; PFR, plug flow reactor.

The initial control fed‐batch experiments in a one‐compartment STR reactor (STR only) evaluated the baseline response of *C. glutamicum* DM1945x3. The data obtained in a well‐mixed homogeneous environment formed the basis from which comparisons were made. The simulations investigated can be broadly divided into four separate studies, as shown in Table 1.

SDR 1–3 simulations were used to investigate the effect of oxygen limitation on *C. glutamicum* DM1945x3 fed‐batch fermentation. SDR 4–6 studied the effect of high glucose concentration in an oxygen‐limited environment. SDR 7–9 investigated how the bacteria cells responded to high pH oscillations in an oxygen‐limited surrounding. Finally, SDR 10–14 observed the combined effect of high glucose and pH gradients in an oxygen‐limited environment on the growing bacterial cells. SDR 10–12 were conducted using the current protocol of constant *f*_m_ whilst SDR 13 and SDR 14 followed the usual literature protocol in that the volume in the STR was not decreased as the *τ*_PFR_ was increased. This was done to directly compare the new approach (runs SDR 10–12) to the more usual approach of constant volume (SDR 10, SDR 13 and SDR 14) with respect to the impact of these two scale‐down strategies on cadaverine production on scale‐up. The different addition points and average cell residence times in relation to each of these simulations are also highlighted in Table 1.

**Figure 3:**
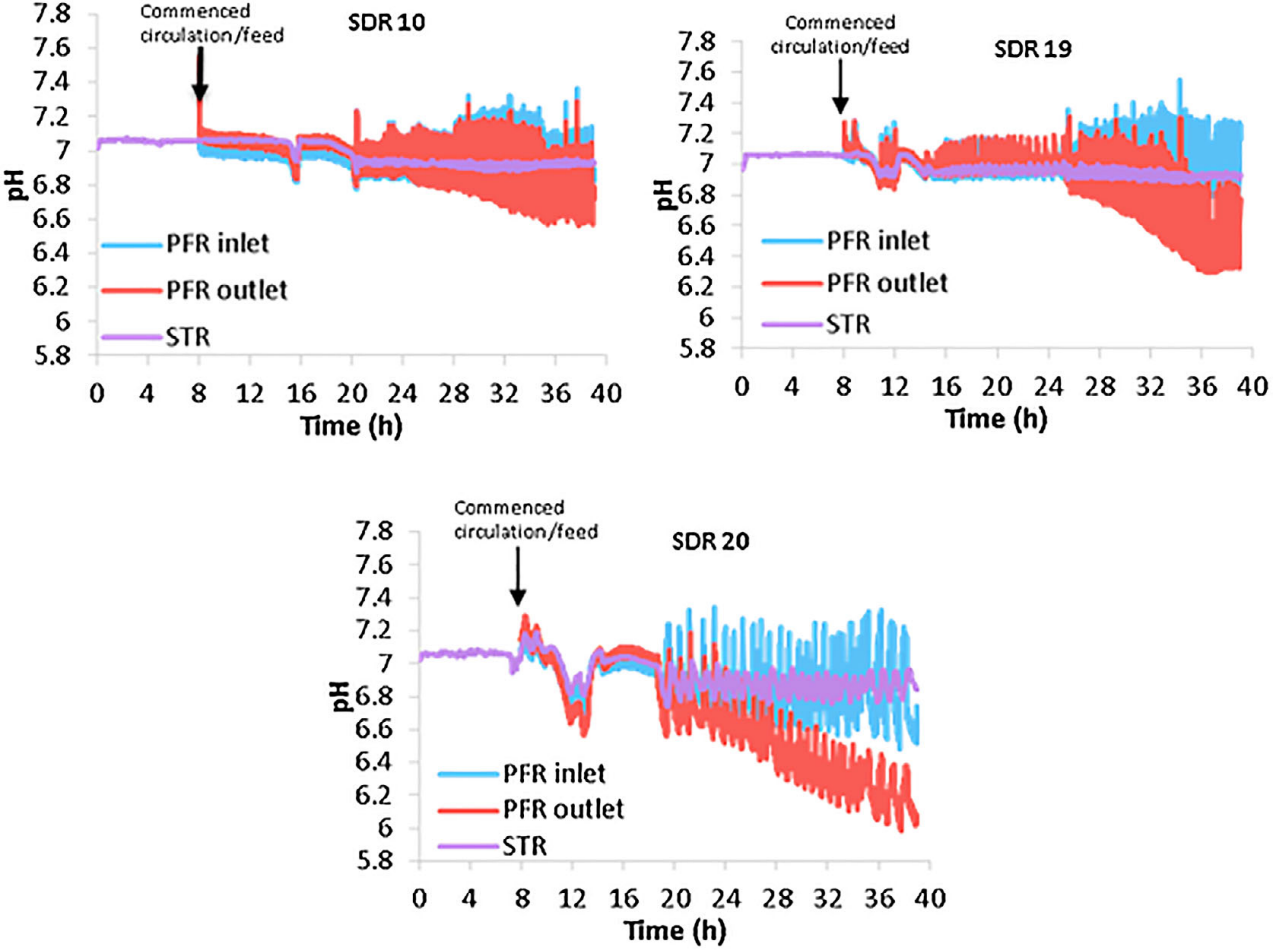
The pH profile across the scale‐down reactor (SDR) as the *τ*_PFR_ was increased from 1 to 5 min

Figure 3 gives an overview of how the pH profile changed with increasing magnitude of fermentation gradients. The difference in pH values at the PFR inlet to the outlet became more significant as the *τ*_PFR_ was increased, hence confirming that the experience of the cells in the PFR was substantially different from that in the well‐mixed STR. Although the volumetric proportion of PFR to STR was in some cases high, especially in simulations with the *τ*_PFR_ of 5 min, the conditions were set to mimic the most extreme gradients in a large‐scale high cell density fed‐batch fermentation and to emphasize the differences that can occur with the two protocols. In addition, by using a wide range of simulated conditions, a better indication could be obtained of *C. glutamicum'*s sensitivity of metabolite production changes with the increasing degree of heterogeneity found on scale‐up. Thus, this approach gives a process tool which would indicate metabolite thresholds that need to be monitored and the occurrence of mixing inefficiencies during fermentation scale‐up.

**Figure 4:**
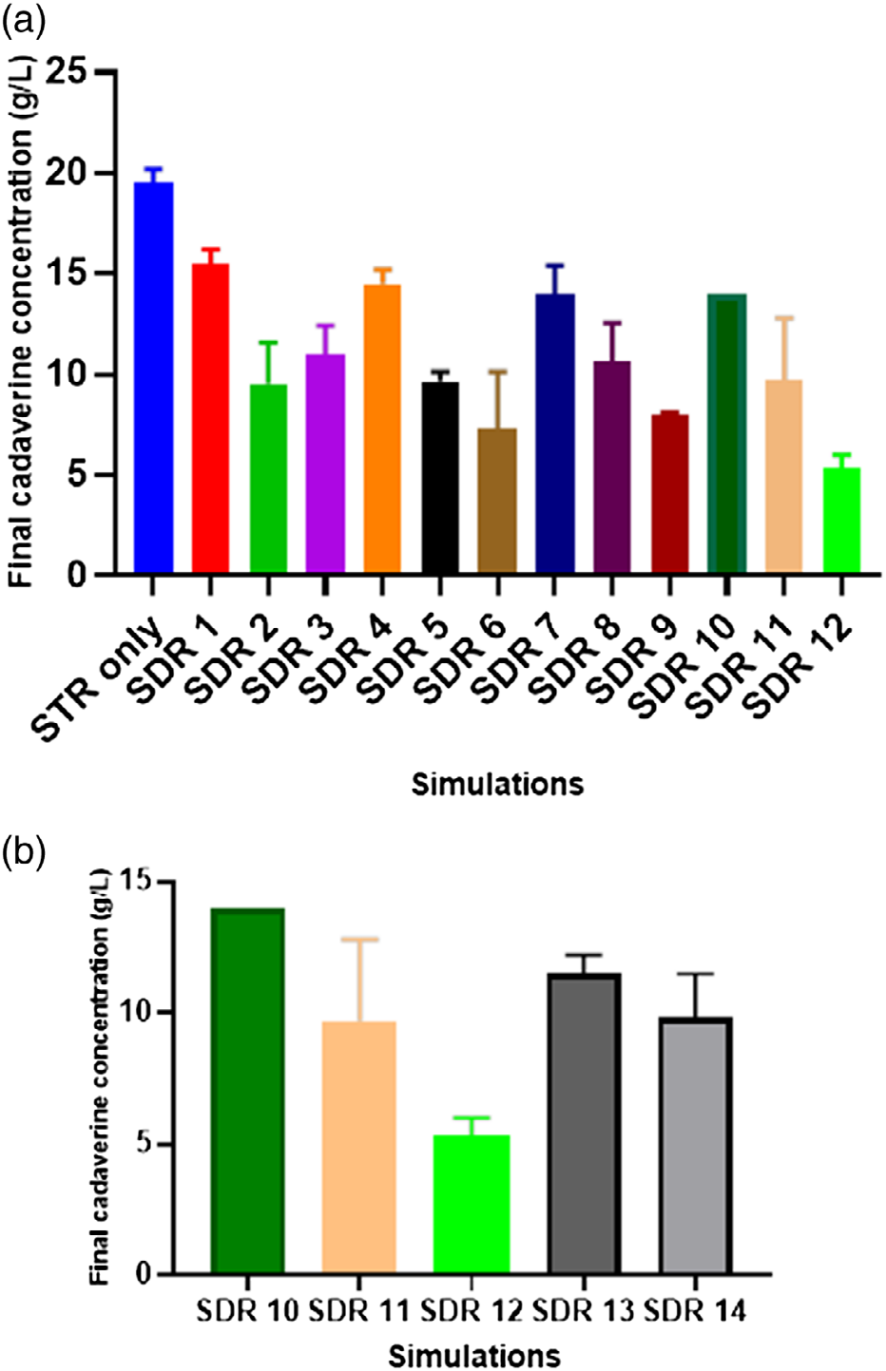
(a) Final cadaverine concentrations (at 39 h) of the respective scale‐down reactor (SDR) simulations investigated. (b) Effect of two scale‐down strategies on final cadaverine concentrations (at 39 h), SDR 10, SDR 11 and SDR 12 – the current scale‐down strategy, *τ*_PFR_ increased from 1 to 5 min and *f*_m_ constant at 0.0018 s^−1^, SDR 10, SDR 13 and SDR 14 – conventional scale‐down strategy *τ*_PFR_ increased from 1 to 5 min and *f*_m_ varied from 0.0018, 0.0009 to 0.0004 s^−1^. The error bars indicate the standard deviations of two biological replicates

The main product of interest was cadaverine, and its importance as a precursor in the manufacture of novel materials for the biorefinery of the future is well known.^23^, ^24^, ^25^ Intracellularly, the production of cadaverine depends on its central precursor l‐aspartate, which is derived from one tricarboxylic acid (TCA) cycle intermediate – oxaloacetate.^26^ The highest concentration of cadaverine (≈19.5 g L^−1^) produced was seen in the control fermentation STR only, highlighted in Fig. 4. In contrast, the lowest amount of cadaverine (on average = 5.3 g L^−1^, a 73% decrease compared to STR only) formed was seen in SDR 12 (deemed to be the harshest simulated fermentation environment).

The results from Fig. 4 show that the final cadaverine titre was dependent on the magnitude of the fermentation gradients simulated. The trend seen indicates that the more the fermentation gradients predominate, the more significant the decline in the amount of cadaverine produced. This loss in cadaverine titre implies that the harsh environment simulated by the SDR introduces an added constraint on *C. glutamicum* DM1945x3 productivity. Thus, in response to the harsh PFR environment, these bacterial cells most likely activated alternative metabolic pathways to promote survival, a situation which may have led to the observed losses in cadaverine concentrations of the SDRs fermentations.

Figure 4 also indicates that the longer the *τ*_PFR_ the less cadaverine produced; this trend was predominant across each of the four main divisions of the simulations studied. At a *τ*_PFR_ of 1 min, the final cadaverine accumulated was similar across SDR 1, SDR 4, SDR 7 and SDR 10 (see Fig. 4), on average, this was evaluated at 14.5 g L^−1^. One inherent assumption here is that the *τ*_PFR_ of these SDRs roughly equate to the mixing times in large‐scale fermenters (> 12 m^3^, mixing time, 1–4 min).^12^

One insight from this work shows that, if this fed‐batch fermentation process is scaled‐up in its current form with a mixing time of 1 min, roughly 28% loss in cadaverine is predicted regardless of where the addition zone is located.

The effect of adopting the new scale‐down strategy used here compared to the conventional scale‐down strategy can be seen in Fig. 4(b). It highlights how cadaverine productivity was affected with the current strategy (SDR 10–12) compared to similar simulations (SDR 10, SDR 13 and SDR 14) using the conventional scale‐down strategy,^16^, ^27^, ^28^ where only the *τ*_PFR_ was varied without consideration for *f*_m_. This comparison suggests that increasing only *τ*_PFR_ does not necessarily translate to a high productivity loss, as seen in Fig. 4(b) (SDR 10, SDR 13 and SDR 14) when the *τ*_PFR_ was increased from 1 to 2 min and then to 5 min, with a concurrent decrease in *f*_m_ from 0.0014 to 0.0009 to 0.0004 s^−1^,respectively. Here the loss with increasing residence time in the PFR is a lot less (∼14 gL^−1^ down to ∼9.8 gL^−1^) than in the equivalent constant *f*_m_ approach (STR 10 with ∼14 gL^−1^ to STR 12 with ∼5.3 gL^−1^). This difference in loss of productivity is probably because, in the traditional approach in SDR 13 and SDR 14, the cells had more time to recover in the well‐mixed STR in contrast to SDR 11 and SDR 12. Overall, the cadaverine loss at the *τ*_PFR_ of 5 min in the traditional approach was only about 30%, whilst with the current approach it was 62% compared to STR 10. This result would suggest that the scale‐down strategy adopted here is more likely to indicate the impact on productivity of a worsening mixing situation as a fed‐batch fermentation is scaled‐up.

The decrease in cadaverine concentration illustrated in Fig. 4 is most likely due to the activation of the TCA cycle reductive pathway, overflow metabolic pathway, energy expended in intracellular pH homeostasis and the metabolic shift to the fermentative pathway in response to the oxygen limitation and high pH/glucose environment of the PFR. These alternative pathways act as energy sinks which form the basis for the depletion of precursor compounds (for example, oxaloacetate) used for the formation of cadaverine.

The exposure of *C. glutamicum* DM1945x3 cells to the oxygen limitation in the PFR had a detrimental effect on cadaverine productivity (seen in all SDRs here). Although *C. glutamicum* is a facultative anaerobe, its survival in a low oxygen environment comes at a cost to productivity. This adaptation occurs by upregulating its malate dehydrogenase enzyme activity within the TCA cycle,^29^ an enzyme known to reduce oxaloacetate to succinic acid. This competition reaction reduces the pool of oxaloacetate available for cadaverine production. Also, the second metabolic shift is the increase in the conversion of pyruvate to lactic and acetic acid by the action of the lactate dehydrogenase, pyruvate: quinone oxidoreductase and the CoA transferase A enzymes.^30^ This consequently reduces the carbon flux to the TCA cycle. Thus, the interaction of activating both the TCA cycle reductive and fermentative pathway contributed to the cadaverine losses seen in all SDRs, but solely pertinent in the case of SDR 1–3.

The high glucose environment of the oxygen‐limited PFR section of SDR 4–6 most probably activated the overflow metabolic pathway. This occurs as a result of the increased carbon flux from glycolysis saturating the glyceraldehyde‐3‐phosphate dehydrogenase and the pyruvate dehydrogenase enzymes. This metabolic bottleneck promotes the diversion of excess carbon molecules to the production of dihydroxyacetone and lactate, which ultimately reduces the carbon flux to the TCA cycle and cadaverine formation.^17^

The pH gradients induced in SDR 7–9 were in the range from 6.2 to 7.6. However, it is known that *C. glutamicum* can tightly regulate its optimum intracellular pH of 7.5 within an external pH range 7–8.5.^31^ Thus, the more the extracellular pH deviates from the optimum, the more energy *C. glutamicum* cells use in maintaining a constant internal pH. Its pH homoeostasis is controlled by the dynamic influx and efflux of ions across the cytoplasmic membrane. Thus, when a cell fluctuates its membrane potential as a response to the external environment, it reduces the amount of adenosine triphosphate (ATP) (the energy currency for cell metabolism) available for other cellular functions. In an acidic medium (as observed in the uncontrolled pH environment of the PFR), *C. glutamicum* also triggers an iron starvation response by suppressing iron‐containing enzymes, (such as catalase, succinate dehydrogenase, and aconitase) which consequently reduces the pool of oxaloacetate in the TCA cycle and activates the competitive methionine pathway.^31^ This indicates that the exposure of *C. glutamicum* DM1945x3 to a continually changing pH range outside its optimum within the PFR section was most likely a major contributing factor to the loss in cadaverine production for these simulation studies.

For SDR 10–12, the culminating effect of exposing *C. glutamicum* DM1945x3 to oxygen limitation, pH and glucose gradients of the PFR, as discussed earlier, most probably led to the even more significant losses in cadaverine seen in these simulations.

**Figure 5:**
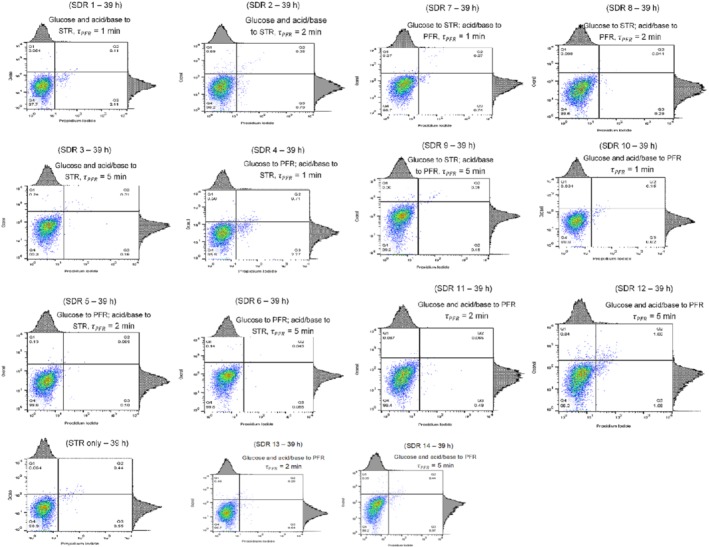
Flow cytometer dot plots for scale‐down reactor (SDR) 1–14 and stirred tank reactor (STR) only indicating the condition of the cell membrane at the end of fermentation. Each dot on the plot represents a cell of the 10 000 *Corynebacterium glutamicum* DM1945x3 cells interrogated

The flow cytometry analyses clearly distinguished three cytoplasmic membrane states illustrated in the four quadrants of Fig. 5 for SDR 1–14 and STR only fermentations. Quadrants Q4, Q1 and Q2 represent healthy, depolarized and permeabilized (dead) cells respectively. Q3 most likely indicates cell clusters and/or equipment noise. In developing a flow cytometer multi‐stain assay for *C. glutamicum*, its unique outer membrane structure was taken into consideration. This is because its outer layer is composed of mycolates with similar functions to that of a Gram‐negative organism.^32^ This results in an unusually high resting membrane potential of ≈170 mV in a neutral medium, uncharacteristic of a Gram‐positive microbe.^33^ Thus, the fluorochromes (propidium iodide and Oxonol) used here are typical for the analysis of Gram‐negative microorganisms.

Figure 5 shows that in all cases, the predominant cell subpopulations were cells with an intact cytoplasmic membrane – Q4 (deemed healthy). At the end of the fermentation, the Q4 quadrant ranged from 96% to 99% of the total cell population. In some of the SDRs such as SDR 4 and SDR 12, a significant Q3 subpopulation was observed (2–3%). This subpopulation was most likely due to the presence of cell clusters (healthy and dead cells).^34^, ^35^ As such, the Q3 subpopulation occurs when a dead cell stained by the PI and oxonol fluorochromes attaches to an unstained healthy cell, resulting in the false‐negative for oxonol as seen in SDR 4 and SDR 12 of Fig. 5. This propensity for formation of doublets is closely linked to *C. glutamicum's* cells V‐shaped morphology.^36^ However, from these results, there was no correlation between the presence of Q3 and the fermentation gradients simulated. In all, the results of Fig. 5 show that increasing the magnitude of fermentation gradients had no significant effect on *C. glutamicum* DM1945x3 membrane integrity.

**Figure 6:**
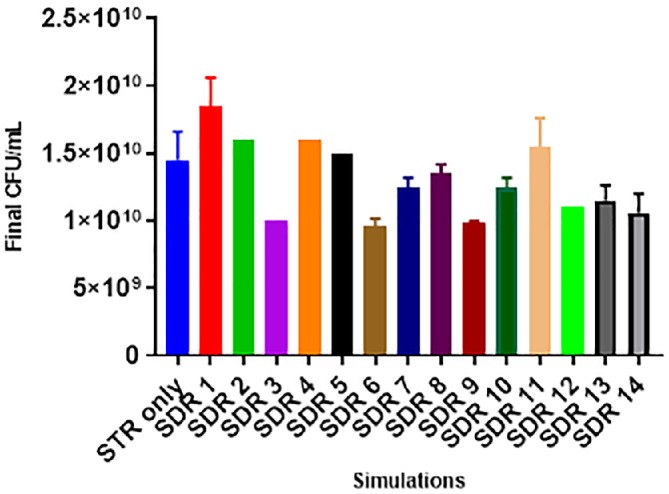
Final colony‐forming unit (CFU) counts (at 39 h) of the respective simulations investigated, the error bars indicate the standard deviations of two biological replicates

The CFU profiles indicate that the bacterial cells ability to form colonies on TSA plates declined, especially in simulations when the *τ*_PFR_ = 5 min (Fig. 6). This suggests that in fermentations with the highest level of gradients (pH, glucose and DO), which occurred at the *τ*_PFR_ of 5 min affected the formation of visible colonies. This is seen in the low CFU values of SDR 3, SDR 6, SDR 9, SDR 12 and SDR 14 they all showed the lowest cell count, and when compared to the control STR only on average, these losses were 31%, 34%, 31%, 24% and 27% respectively (Fig. 6).

Combining the flow cytometry and CFU results may suggest that the number of viable but non‐culturable cells increased as the magnitude of fermentation gradients goes above a threshold. This is because the flow cytometer analyses illustrated that in all simulations, the cells were predominantly healthy (≥96%). However, the CFU count results indicate that cells with an intact membrane do not necessarily correlate positively to forming visible colonies. It could thus be inferred that the quality of *C. glutamicum* DM1945x3 (concerning viable colony formation) declined as the degree of fermentation gradients increased.

In all fermentations here, the cell growth rate was controlled by limiting the amount of glucose added to the fermenter, as stipulated by Eqn (3). The pump feeding flow rate was automatically regulated by the Biostat B‐Plus controller and set such that it adhered to a low *μ* of 0.1 h^−1^. This *μ* of 0.1 h^−1^ was adopted to ensure that the production of side products was kept to a minimum.^27^

**Figure 7:**
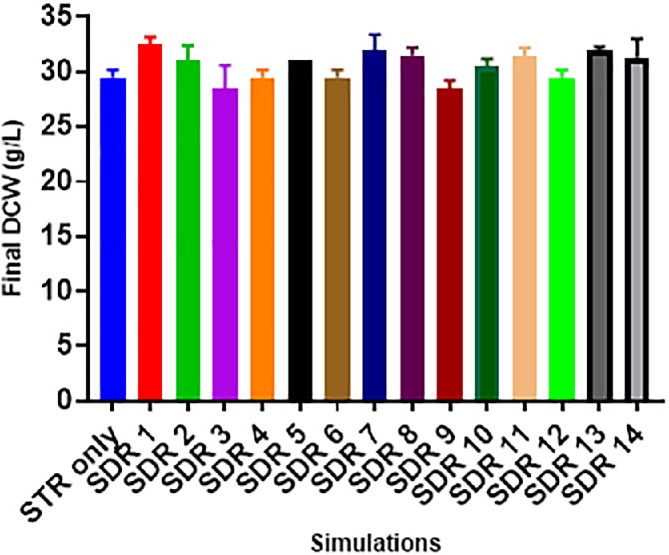
Final dry cell weight (DCW) concentrations (at 39 h) of the respective simulations investigated, the error bars indicate the standard deviations of two biological replicates

The cell growth was monitored by quantifying the DCW change in time. The DCW gives the total biomass concentration (viable and non‐viable cells) in the fermenter. Figure 7 suggests that the DCW was not affected in any of the SDRs studied. These results broadly agree with other findings which portray *C. glutamicum* as a resilient organism that adapts easily to fermentation gradients.^21^, ^28^, ^37^ However, it is essential to restate the detrimental effect of increasing fermentation gradients as shown in Fig. 4, which would make scaling up with this current strain uneconomical. This calls into question the supposed robustness of *C. glutamicum* as an industrial microorganism when faced with a very wide range of simulated heterogeneous conditions.

The insignificant effect to the final DCW could be attributed to *C. glutamicum* DM1945x3 inherent efficient ability to adapt its energy generation pathway for replication. For example, in a limited oxygen environment, *C. glutamicum* favours the synthesis of biomass via its lower glycolysis or/and oxidative phosphorylation pathways, while energy generation from the TCA cycle is suppressed.^38^ This ability to continuously activate and reactivate different energy generation pathways for biomass production in a heterogeneous environment most likely gives it the level of resilience seen from Fig. 7. This characteristic sets it apart from organisms such as *E. coli*, which do not have this level of adaptability and are much more affected by fermentation gradients.^27^, ^39^

**Figure 8:**
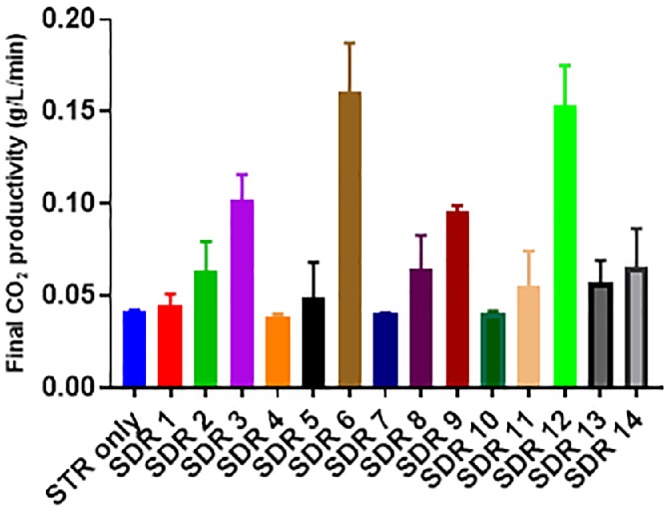
Final carbon dioxide (CO_2_) productivity (39 h) of all simulations investigated compared to stirred tank reactor (STR) only; the error bars indicate the standard deviations of two biological replicates

The production of CO_2_ is strongly linked to the growth rate and metabolic pathway(s) of a bacterial cell. For example, if two microorganisms with the same growth rate have different metabolic pathways to ATP production, their CO_2_ production rate will vary. This is because of the organism‐specific number of intermediate oxido‐redox reactions needed for the production of ATP.^40^ Figure 8 shows the final CO_2_ productivity of all cases investigated.

The general trend seen in Fig. 8 indicates a positive correlation between *τ*_PFR_ > 1 min and CO_2_ productivity. There was no difference observed in CO_2_ productivity when SDR 1, SDR 4, SDR 7 and SDR 10 were compared to the STR only. However, when the *τ*_PFR_ was increased to 2 min; the difference in CO_2_ productivity compared to STR only then became significant. On average, the increase in CO_2_ productivity compared to STR only at the *τ*_PFR_ of 2 and 5 min was 1.4‐fold and 3.1‐fold, respectively. However, SDR 14 (simulated using the traditional scale‐down strategy) was in contrast to the other simulations here with the *τ*_PFR_ of 5 min due to the increased chance of the cells having more time to recover in the ideal well‐mixed STR environment. This effect is confirmed in Table 1, where SDR 14 was shown to have the longest τ_STR_ of 50 min. Of all the simulations, SDR 6 and SDR 12 produced the most CO_2_ (0.16 and 0.15 g L^−1^ min^−1^, respectively); this similarity in value is linked to their common glucose addition point. The addition of a highly concentrated glucose (620 g L^−1^) feed introduced in the oxygen‐limited PFR, as simulated in SDR 6 and SDR 12, promotes the activation of both the fermentative and overflow pathways for ATP production. CO_2_ is known to be a major side product of these pathways, hence the high level of productivity observed in SDR 6 and SDR 12.^30^, ^41^

Also, from Figs 4 and 8, it can be inferred that there is a negative correlation between CO_2_ productivity and cadaverine production. This suggests the predominance of wasteful metabolic pathways as the cell responded to the harsh SDR environment.

## CONCLUSION

The ability to simulate spatial and temporal gradients of the large‐scale fermenter in the laboratory is both a convenient and economical method for characterizing biological processes and predicting fermentation performance. A new scale down protocol was introduced in which the mean frequency at which the cells entered the PFR (*f*_m_) was kept constant. This was achieved by reducing the volume in the STR as the *τ*_PFR_ was increased. This approach made the fermentation gradients (DO, pH and glucose concentrations) in the PFR more pronounced, and it also reduced the amount of time the cells spent in the well‐mixed STR during which they might be able to recover from the impact of the changing environment in the PFR. This approach was shown to be a more sensitive indicator of loss of productivity on scale‐up due to loss of homogeneity associated with poorer mixing than the traditional scale‐down approach.

The results here confirm that the cell physiological response is a function of its microenvironment. Hence, the different degree of losses in fermentation productivity was linked to the design of the SDR simulated. The most significant decline in cadaverine productivity was seen in the SDRs with the longest *τ*_PFR_ of 5 min. The interactions of a localized high glucose concentration, high pH oscillations and a limited oxygen environment, with an increased *τ*_PFR_, were the major contributors to the decrease in cadaverine productivity observed. The flow cytometry and CFU analyses suggested the predominance of viable but non‐culturable bacterial cells as the fermentation gradients increased. However, the biomass production was not affected in any of the SDRs studied. CO_2_ productivity generally increased as the degree of fermentation gradients increased.

This study indicates that the *C. glutamicum* DM1945x3 did respond to its changing environment to survive, and in doing so, expending energy to maintain intracellular homoeostasis at the detriment of cadaverine productivity. Thus, if this process were to be scaled up in an STR, losses are to be expected due to increasing spatial and temporal heterogeneities.

## AUTHORS STATEMENT

This work is part of the project “SCILS ‐ Systematic consideration of inhomogeneity at the large scale,” which was embedded within the ERA‐IB2 framework (EIB.12.057), funded by BBSRC grant ref BB/L001284/1, which the authors gratefully acknowledge (see https://dspace.lboro.ac.uk/2134/34284). In addition, W. Olughu received a PhD studentship co‐funded by EPSRC DTA and Loughborough University.
